# Comparison of Two Frailty Assessment Methods and Their Association with Functionality in Subjects with Exacerbation of COPD

**DOI:** 10.1155/2023/6660984

**Published:** 2023-05-11

**Authors:** Andrea Akemi Morita, Rafaela Furlan Munhoz, Giovana Labegalini Guzzi, Vanessa Suziane Probst

**Affiliations:** ^1^Stricto Sensu Graduate Program in Rehabilitation Science of State University of Londrina and Pitágoras Unopar University, Londrina, Paraná, Brazil; ^2^State University of Londrina, Physiotherapy Department, Londrina, Paraná, Brazil

## Abstract

**Objectives:**

To verify the prevalence of frailty in patients hospitalized with acute exacerbation of COPD; to compare two frailty assessment methods: Edmonton Scale and the Fried Frailty Phenotype, and to associate frailty with functioning in these patients.

**Methods:**

Patients hospitalized due to an acute exacerbation of COPD were included. The assessment of pulmonary function, frailty, and functioning was performed. Frailty assessment was performed by the Edmonton Scale and Fried Frailty Phenotype. Individuals were classified into “frail,” “pre-frail” and “non-frail.” Functioning was evaluated by the one sit-to-stand test.

**Results:**

Thirty-five individuals were included (17 male, 69 ± 9 years; FEV1/FVC 47 ± 10%; FEV1 34 (24–52) % predicted). Participants scored 3 (3-4) points on the Edmonton Scale and 7 (5–9) points on the Fried Frailty Phenotype. According to the Fried model, 17% were considered prefrail and 83% frail and in the Edmonton scale, 20% were classified as nonfrail, 29% prefrail, and 51% frail. There was a positive moderate correlation between the two methods (*r* = 0.42; *p*=0.011); however, there was no agreement between them (*p*=0.20). This probably occurs because they assess the same construct, i.e., frailty; however, they are different in their components. There was a negative and moderate correlation between the Fried Frailty Phenotype and functioning (*r* = −0.43; *p*=0.009).

**Conclusion:**

Most hospitalized individuals with exacerbated COPD with severe and very severe airflow limitation are frail and the assessment methods correlate, but there is no agreement. Additionally, there is association between frailty and functioning in this population.

## 1. Introduction

Subjects with exacerbations of chronic obstructive pulmonary disease (COPD) have worse quality of life and decline in lung function [1]. Exacerbations are associated with mortality, and this has a significant socioeconomic impact on public health policies [2]. In COPD exacerbation, exercise capacity, physical activity, and functional capacity are also impaired [3, 4]. Among all the involved factors, muscle dysfunction, caused by physical deconditioning, poor nutrition, and myopathy contributes to exercise intolerance and impaired functioning [5]. This reduced functioning and increased dependence can also be reflected in frailty in these patients [6].

Frailty is a biological syndrome of reduced reserve and resistance of several physiological systems [7]. It is more prevalent with increasing age and is related to high risk to health possibly leading to mortality, institutionalization, falls, and hospitalization [8]. It is known that elderly with COPD are more likely to be frail than healthy elderly [9–11], and the majority of patients with COPD are considered frail, which increases the risk of exacerbation and hospitalizations [12, 13]. In addition, COPD severity and symptoms like dyspnea induce inactivity, which leads to loss of muscle strength, immobility, and frailty [12, 14].

Several frailty assessment instruments are validated in the COPD population such as Fried Fragility Phenotype, FRAIL Scale, Edmonton Scale, and Deficit Accumulation Index (DAI) [14, 15]. There are some studies that compared these instruments [16, 17]; however, there is no evidence of this comparison in patients with exacerbation of COPD. Hence, the objectives of this study were to verify the prevalence of frailty in patients hospitalized with acute exacerbation of COPD; to compare two frailty assessment methods: Edmonton Scale and the Fried Frailty Phenotype. Secondarily, we aimed to associate frailty with functioning in these patients. These two instruments were chosen, among many others, because Fried Frailty Phenotype is already widely used and validated in the literature and the Edmonton Scale because it has been employed in a hospital environment [14]. Furthermore, the Fried Frailty Phenotype scale was used as reference to verify the concurrent validity of frailty assessment.

## 2. Methods

This is a cross-sectional and observational study, with a convenience sample of patients with COPD, hospitalized for at least 72 hours, due to an acute exacerbation of the disease. The study was conducted at the University Hospital of Londrina, in the State University of Londrina and Zona Sul Hospital, in Londrina, Paraná, Brazil.

The inclusion criteria were patients diagnosed with COPD according to the Global Chronic Obstructive Lung Disease [18]; admitted to the hospital due to exacerbation of COPD; in spontaneous ventilation, without the need of mechanical ventilation; with absence of important comorbidities that could interfere in the performance of physical tests; and without history of hospitalization due to exacerbation of the disease in the previous month. Any patient who did not demonstrate physical or cognitive conditions to perform the tests, gave up to participate in the study for any reason, died or had respiratory failure, and required mechanical ventilation assistance was excluded from the study.

This study was approved by the Research Ethics Committee of the State University of Londrina (2.782.671). All participants gave written consent to participate.

Subjects performed during hospitalization, an evaluation of pulmonary function, i.e., spirometry, to confirm the diagnosis of COPD. In the first 72 hours of hospitalization, anthropometric data were collected and frailty was assessed using the Fried Frailty Phenotype and the Edmonton Frailty Scale. In addition, functioning was also evaluated by the timed Up and Go (TUG) and sit-to-stand (STS) tests.

### 2.1. Assessment

Pulmonary function: Spirometry was performed with a portable spirometer. Patients carried out the test according to international guidelines [19, 20], and Brazilian reference values were used for analysis [21].

Body weight and height: Body weight and height of each patient were assessed and the body mass index (BMI) was calculated.

Sit-to-stand (STS): The test was performed using a chair, following the protocol of Ozalevli et al. [22] The STS is valid and reliable [23]. The subjects were instructed to remain seated, with their arms crossed over their chest, and to perform the movement of sitting down and standing up as many time as possible within 1 minute. The number of repetitions that the patient performed within 1 minute was the outcome. Reference values according to gender and age were used [24].

### 2.2. Frailty

The Fried Frailty Phenotype and the Edmonton Scale were used to assess frailty. The Fried Frailty Phenotype [7] is composed by 5 criteria, and among them, there were unintentional weight loss, fatigue, walking speed, handgrip strength, and physical activity of daily living (PADL). The score ranges from 0 to 5, with the individual scoring 0 being considered nonfrail, 1-2 prefrail and 3–5 frail.

Unintentional weight loss: patients were asked if they lost more than 4.5 kg in the last year, if the answer was “yes,” it would indicate frailty.

Handgrip strength: a hand held dynamometer was used and patients were instructed to sit on a chair, with elbows flexed at 90° and forearm in a neutral position, without upper limb support. Then, they were asked to squeeze the equipment as hard as possible, and the peak force was considered. The maneuver was repeated 3 times on each limb and the highest value of each was taken into account. Scoring was performed according to gender (men or women), BMI (less than 24, 24.1 to 26, 26.1 to 28, more than 28 kg/m^2^), and grip strength cutoff (for men 29 kg to 32 kg and for women 17 to 21 kg) [25].

Fatigue: two questions from the Center for Epidemiological Studies - Depression (CES - D) scale were used to check for fatigue, as follows: “I felt that everything I did was an effort” and “I could not get going.” “How often in the last week did you feel this way?.” The response options included: 0: rarely (˂1 day), 1: some or a little of the time (1–2 days), 2: a moderate amount of the time (3–4 days), or 3: most of the time. If they answered “2” or “3” to either of these questions, they were classified as frail according to the fatigue criterion [26].

Walking speed: Patients needed to walk in a 4-meter corridor at their usual pace and their walking speed was evaluated. Scoring was done according to gender (men or women), height (men less or more than 173 cm and women less or more than 159 cm), and time to walk (more or equal to 7 seconds and less or equal to 6 seconds). The cutoff used was proposed by Fried et al. [7].

Physical activity of daily living: Patients worn a physical activity monitor (Actigraph wGT3X-BT®, Actigraph Corporation, USA) for at least 2 consecutive days, for 24 hours, which allows checking their daily energy expenditure. The device was positioned on the waist line and on the midline of the right thigh and should be removed only for bathing or water activities [12]. Kilocalories (kcal) were calculated using a standardized algorithm stratified by sex [27]. Men who had less than 383 kilocalories per week and women who had less than 270 kilocalories per week were classified as frail [7].

The Edmonton Frailty Scale [14, 28] is a subjective assessment method that is composed by 9 domains: health status, cognition, nutrition, medications, social support, functional independence, mood, continence, and functional performance (assessed using the Timed Up and Go test - TUG). Its score ranges from 0 to 17, in which 0–4 the subject is classified as not frail, 5-6 apparently vulnerable, 7-8 mild frailty, 9-10 moderate frailty, and 11–17 severe frailty [13]. All domains are assessed through questions in which the answers must be “yes” or “no,” and “yes” was considered frailty [13].

Timed Up and Go test (TUG): The test was performed according to the protocol proposed by Podsiadlo and Richards [29], and it is valid and reliable [23], in which the patient was instructed to get up from a chair, walk in a straight line of 3 meters marked on the floor at a comfortable and safe pace, turn around, walk back to the chair, and sit down. Two tests were performed, and the best performance was used for analysis. TUG time >11 seconds was considered functional impairment [30].

### 2.3. Statistical Analysis

Statistical analysis was performed using the GraphPad Prism 6.0 (GraphPad Software Inc., San Diego, California, USA) and the SPSS 20.0 (Statistical Package for the Social Sciences Inc., Chicago, Illinois, USA) software. The Shapiro–Wilk test was used to verify data distribution and according to the data normality; results were described as mean and standard deviation or median (interquartile range 25–75%).

The Kappa test was used to analyze the agreement between the two methods. The proportion of individuals classified as nonfrail, prefrail, and frail according to each instrument was evaluated using the Chi-square test.

Moreover, to verify the correlation between the two frailty assessment methods and between frailty and functioning, the Spearman's Correlation Coefficient was used. The statistical significance set for all analyses was *p* < 0.05.

The power of the study was calculated retrospectively (GPower 3.1, Heinrich-Heine-University, Dusseldorf, Germany). Based on the correlation between the Fried Frailty Phenotype and Edmonton Frailty Scale of *r* = 0.42, considering an *α* of 0.05, the sample size has a power of 82%.

## 3. Results

Forty patients with acute exacerbation of COPD were included in the study. However, five were excluded after entry due to the following reasons: mental confusion and difficulty in performing the proposed tests. Therefore, data from 35 patients were analyzed and their characteristics are shown in Table 1. In general, patients were elderly, in a similar proportion of men and women (*p*=0.73), overweight and with moderate to severe airflow obstruction.

The functional profile presented in Table 2 shows that patients had impaired walking speed. Twenty-one individuals (60%) also presented reduced handgrip strength. All subjects presented impairment in the STS test and 85% had mobility impairment assessed by TUG.

**Figure 1:**
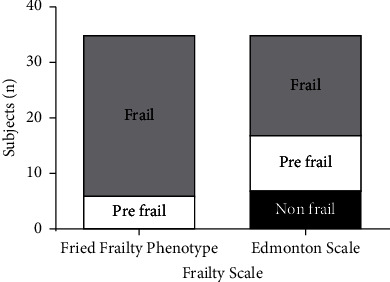
Frailty classification according to Fried Frailty Phenotype and Edmonton Scale frailty scale.

Most of the individuals were considered frail according to the two assessment instruments (Table 1). The proportion of individuals classified as non-frail, prefrail and frail was different on the Edmonton and Fried Frailty Phenotype (*p*=0.005), with the majority being classified as frail according to the Fried Phenotype (Figure 1).

**Figure 2:**
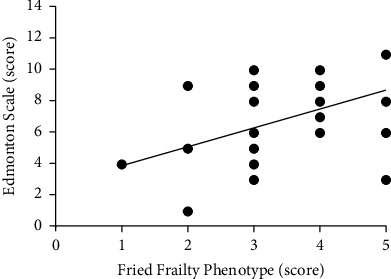
Correlation between Edmonton Scale and Fried Frailty Phenotype (*r* = 0.42; *p*=0.011).

There was a positive and moderate correlation between the Fried Frail Phenotype score and the Edmonton Scale (*r* = 0.42; *p*=0.011) as shown in Figure 2. However, there was no agreement between the two instruments (*p*=0.20).

**Figure 3:**
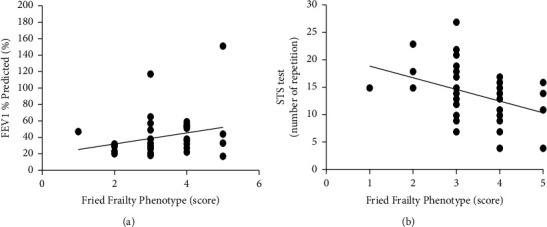
Correlation between score in Fried Frailty Phenotype and number of repetitions in the sit-to-stand test (a) (*p*=0.009) and FEV1% predicted (b) (*p* > 0.05).

**Figure 4:**
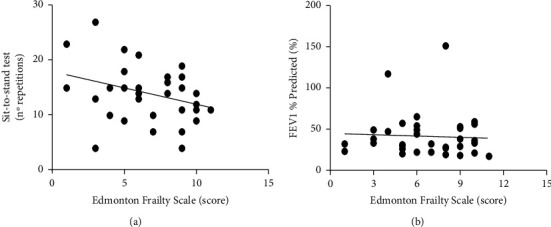
Correlation between score in Edmonton Frailty Scale and number of repetitions in the sit-to-stand test (a) and FEV1% predicted (b) (*p* > 0.05 for all).

When the frailty assessed by two instruments was correlated, it was found an association with functional outcomes. It was possible to verify a negative and moderate correlation only with Fried Frailty Phenotype and the sit-to-stand test in number of repetitions (*r* = −0.43; *p*=0.009) (Figure 3). There was no correlation with any other functioning outcomes. The frailty instruments did not correlate with pulmonary function variables and COPD severity (*p* > 0.05 for all) (Figures 3 and 4).

## 4. Discussion

This study found that the majority of patients hospitalized for an exacerbation of COPD is classified with severe and very severe airflow limitation are frail. Furthermore, it was observed that the two frailty assessment instruments correlate but do not agree with each other. Finally, the two instruments are moderately and negatively correlated with functioning.

It is known that frailty in patients with COPD is associated with longer duration of hospitalization and poor quality of life [31]. This population has twofold increase in developing frailty than those without COPD. When people with and without respiratory impairment were compared, it was shown that those with respiratory impairment had 58% increase in the odds of developing frailty at the 3-year follow-up [14]. The prevalence of frailty in patients with COPD among previous studies varied from 9% to 64%. On the other hand, the prevalence of prefrail patients is more uniform and varies from 48% a 64% [14]. In the present study, the prevalence of frailty was high according to the two instruments. In the Fried Frailty Phenotype method, 81% was classified as frail and according to Edmonton Scale, 51%. The high number of frail patients is probably related to their health status, since subjects were evaluated during a hospitalization due to an exacerbation of their disease.

In the majority of previous studies, frailty was analyzed in a stable setting and there are few studies that evaluated frailty in exacerbated and hospitalized COPD patients [32–34]. The prevalence of frailty in a stable COPD condition is well established in the current literature and we know that hospitalization can worse this condition in AECOPD. So that, we believe that frailty scores can improve in a stable condition. And our hypothesis is that the Fried Frailty Phenotype could classify more frailty patients than Edmonton, in stable condition, since the Fried instrument is composed by physical function tasks and it could screen patients with physical limitation. Valenza et al. demonstrated that physical activity can predict the absence or presence of frailty in stable and exacerbated patients with COPD [32]. Bernabeu–Mora et al. assessed frailty in hospitalized patients with COPD with the Edmonton scale and verified that frail subjects have early chance to hospital readmission [33]. Even though these previous studies investigated frailty in subjects with acute exacerbation of COPD in a hospital environment, the objectives were different from the present study, in which we aimed at assessing frailty cross-sectionally using two different instruments during hospitalization of COPD exacerbation.

Several methods have been used to evaluate frailty in COPD patients, such as the Fried Frailty Phenotype, the Frailty Staging System, the Gobbens Frailty Model, the Tilburg Frailty Indicator, the Reported Edmonton Frailty Scale, the Kihon Checklist, and the Functional Geriatric Evaluation [14]. To the best of our knowledge, there is no study which compared these instruments during an exacerbation of COPD. Two different studies found reasonable to moderate agreement between the Fried Frailty Phenotype and the Edmonton Scale, which differs from the present study [35, 36]. This may be related to the population studied, since the authors investigated hospitalized elderly individuals who had chronic diseases in general, not only COPD. However, Nguyen et al. verified a slightly higher prevalence of frailty according to Fried Frailty Phenotype in comparison to the Edmonton Scale [35], as in the present study.

Some studies conducted with healthy elderly have compared more than two frailty models and found similarity to predict mortality and adverse outcomes [16, 17]. The results in the present study were different, since there was no agreement between the two studied instruments. However, while the previous studies investigated which method to assess frailty would better predict different outcomes, the aim of the present study was simply to cross-sectionally compare the Fried's model and the Edmonton scale to detect frailty in a group of exacerbated COPD patients. A hypothesis to be raised is that the difference between correlation and agreement is due to the fact that the two instruments evaluate similar variables, thus they would correlate with each other. However, the assessment is performed differently, in Fried Frailty Phenotype most of items are objective with practical tasks and the Edmonton Scale more subjective, e.g., a questionnaire. Thus, although the two instruments aim to evaluate frailty, they do it considering different aspects, and this could justify the lack of agreement between them. It would also explain the difference in the classification of the frailty degrees by the two tools, especially for individuals with a lower degree of fraity.

There is no consensus on which is the best and most reliable method to evaluate frailty, because each instrument, even though they have the same objective, has different variables. The Fried Frailty Phenotype has more physical function tasks to evaluate frailty; otherwise, the Edmonton Scale is composed by questions. Thus, in clinical practice, the appropriate tool will depend on the purpose and population to be studied, the equipment and logistic availability. However, considering the high proportion of frail patients during COPD exacerbation and the deleterious effects already known, it is imperative to evaluate frailty in order to identify those individuals that should be target for treatment. Moreover, the comparison of the two instruments in this study showed that both of them evaluate frailty and could be used to assess this population. However, the Fried Frailty Phenotype better correlated with functioning and, since it is composed by multiple physical function tasks, it could be used as a screening tool to detect frail patients. This result showed the concurrent validity of this study, since it was determined the presence of frailty considering these two frailty assessment methods, the Fried Frailty Phenotype and Edmonton Scale.

This study was the first to investigate the association between frailty and functioning in exacerbated COPD patients, with severe and very severe airflow limitation. It is known that patients with COPD have multisystem deficits that limit their functioning [12]. The majority of the sample presented low functioning according to the performance in the functional tests. This result is expected, since patients with COPD are known for having a decline in functional capacity which is already present on a stable condition [23]. Therefore, it is awaited that this condition would be worsened during an exacerbation of the disease. Torres-Sanchez et al. studied the impact of hospitalization due to exacerbation of COPD on functioning, and they found an important impairment during this period [37]. In addition, in the present study we found moderate correlation between frailty and functioning. This result can be explained by the fact that some variables such as loss of muscle mass and strength and slow gait speed, which are also components of frailty, can affect functioning as well [14]. Although there was correlation between frailty and functioning, the lack of correlation between frailty and pulmonary function was observed. Probably, this fact occurred due to the characteristics of the studied sample, which was mainly composed by subjects with severe and very severe airflow obstruction. Moreover, Scarlata et al. demonstrated that frailty index was a poor predictor of overall lung function [38].

Despite the efforts, this study presents a few limitations that include the fact that the research was conducted in two tertiary hospitals, where the treatment approach is similar. Different results might have been found if the study had taken place in several hospitals. In addition, the sample included hospitalized patients with severe to very severe airflow obstruction. If subjects with milder disease were studied, the results could have been different. Moreover, the subjects that could not be able to perform physical tests have high chances to be frail; however, they were excluded of our sample. Thus, this can be considered a limitation, since we cannot generalize this result for this profile of patient.

In summary, patients with COPD hospitalized for acute exacerbation had severe and very severe airflow limitation and they are frail. Furthermore, the Fried Frailty Phenotype and the Edmonton Scale are frailty assessment tools that correlate, but do not agree with each other. Finally, the Fried Frailty Phenotype model correlates with functioning in this population. Considering that many factors during hospitalization of acute exacerbation of COPD can develop frailty, it is important to assess this outcome to provide an early physical rehabilitation during this period of time.

## Figures and Tables

**Table 1:** Characteristics of the sample.

Variables	*N* = 35
Gender (M/F)	17/18
Age (years)	69 ± 9
BMI (kg/m^2^)	26 (24–28)
FEV_1_/FVC (%)	47 ± 10
FEV_1_ (liters)	0.76 (0.64–1.02)
FEV_1_ (% predicted)	34 (24–52)
FVC (liters)	1.69 (1.28–2.17)
FVC (% predicted)	58 (43–79)
Fried scale	7 (5–9)
Non frail (%)	0 (0)
Pre frail (%)	6 (17)
Frail (%)	29 (83)
Edmonton scale	3 (3-4)
Non frail (%)	7 (20)
Pre frail (%)	10 (29)
Frail (%)	18 (51)

Values were described as mean ± standard deviation or median (interquartile range 25–75%); Shapiro–Wilk test was used to test normality and *p* value >0.05 indicates normal distribution. M: male; F: female; BMI: body mass index; FEV_1_: forced expiratory volume in the first second; FVC: forced vital capacity.

**Table 2:** Functional profile of the studied sample.

Assessment	Performance
Gait speed (m/s)	0.61
Gait speed (%predicted)	76 ± 16
Handgrip force (kg)	22 (18–28)
Handgrip force (%predicted)	96 (82–126)
Sit-to-stand test (number of repetition)	14 ± 5
Sit-to-stand test (%predicted)	43 ± 15
Timed up and go (seconds)	13.22 (12.10–18.56)
Timed up and go (%predicted)	120 (110–169)

Values were described as mean ± standard deviation or median (interquartile range 25–75%); Shapiro–Wilk test was used to test normality and *p* value >0.05 indicates normal distribution.

## Data Availability

The data used to support the findings of this study are included within the article
